# MicroRNAs as diagnostic biomarkers in periodontitis: a systematic review and meta-analysis

**DOI:** 10.1007/s10266-025-01299-8

**Published:** 2026-01-12

**Authors:** Maaz Anwer Memon, Wan Nazatul Shima Shahidan, Noriko Mizusawa, Thirumulu Ponnuraj Kannan, Rizwan Mahmood, Khairul Mohd Fadzli Mustaffa, Usman Ashraf

**Affiliations:** 1 School of Dental Sciences, Universiti Sains Malaysia, Health Campus, Kubang Kerian, 16150 Kelantan, Malaysia; 2 Department of Pharmacology, School of Medical Sciences, Universiti Sains Malaysia, Health Campus, Kubang Kerian, 16150 Kelantan, Malaysia; 3 Department of Oral Bioscience, Institute of Biomedical Sciences, Tokushima University Graduate School, 3-Kuramoto-Cho, Tokushima City, 7708504 Japan; 4 Human Genome Centre, School of Medical Sciences, Universiti Sains Malaysia, Health Campus, Kubang Kerian, 16150 Kelantan, Malaysia; 5 Institute for Research in Molecular Medicine (INFORMM), Universiti Sains Malaysia, Health Campus, Kubang Kerian, 16150 Kelantan, Malaysia

**Keywords:** Biomarker, Humans, miRNA, Periodontal diseases, Periodontitis, Diagnosis

## Abstract

MicroRNAs (miRNAs) are key gene regulators, and their dysregulation is linked to periodontitis. This systematic review and meta-analysis assessed miRNAs consistently associated with periodontitis, focussing on their diagnostic accuracy and potential as biomarkers for early detection. Following PRISMA guidelines (PROSPERO ID: CRD42024520884), electronic searches were conducted in PubMed, Scopus, Web of Science, and Wiley Online Library up to 31 May 2025. The meta-analysis included studies reporting common miRNAs in at least three independent investigations. Diagnostic accuracy was determined by pooled sensitivity, specificity, and area under the hierarchical summary receiver operating characteristic curve (HSROC). Heterogeneity (Cochran’s Q, I^2^, τ^2^), publication bias, influence diagnostics, and leave-one-out analyses were performed. Stratified analyses by specimen type and assay platform were conducted, and predictive values (PPV, NPV) were estimated across plausible prevalence scenarios using a bivariate random-effects Reitsma model in R (version 2025.05.1). Visual outputs, including forest plots, were generated using RevMan (version 5.4). Certainty of evidence was assessed using the GRADE-DTA framework. Of 552 records identified, 32 studies were included in the review, and 14 studies were eligible for quantitative analysis, with miR-146, miR-155, and miR-223 being the most consistently reported. miR-146 and miR-155 showed high diagnostic accuracy, with area under the curve (AUC) values of 0.928 and 0.903, respectively, while miR-223 demonstrated moderate performance (AUC: 0.787). Heterogeneity was substantial for miR-146 and miR-223 and moderate for miR-155. These findings highlight the potential of these miRNAs as non-invasive biomarkers for distinguishing periodontitis from healthy individuals; however, standardised protocols and further validation are necessary for clinical translation.

**Supplementary Information:**

The online version contains supplementary material available at 10.1007/s10266-025-01299-8.

## Introduction

Periodontal diseases are chronic inflammatory conditions affecting the tooth’s supporting structures, including the cementum, periodontal ligament fibres, gingiva, and alveolar bone [1]. With a prevalence estimated between one-fifth and half of the global population, this condition extends beyond oral health, significantly impacting overall well-being [2, 3]. Periodontitis not only leads to tooth loss but also impairs functions such as mastication, aesthetics, and quality of life [4]; it also shares complex associations with systemic conditions, including diabetes mellitus (DM), rheumatoid arthritis (RA), coronary heart disease (CHD), adverse pregnancy outcomes, and pulmonary diseases [2]. Notably, the relationship between periodontitis and these conditions is bidirectional, with emerging evidence indicating that systemic diseases can not only be exacerbated by periodontal inflammation but may also influence the pathophysiology of periodontitis through shared immunoinflammatory pathways [5]. These associations are driven by the translocation of microorganisms and inflammatory mediators from periodontal tissues to distant organs, contributing to systemic inflammation and disease progression [6].

Recent advancements in molecular biology have expanded our understanding of gene regulation and challenged the classical central dogma [7]. It is now recognised that mRNA transcription does not always culminate in protein translation, with various post-transcriptional processes playing critical roles. Among these, epigenetic mechanisms such as DNA methylation, chromatin remodelling, and particularly microRNA (miRNA)-mediated regulation, have garnered significant attention [8, 9]. MiRNAs, short non-coding RNA sequences comprising 18–22 nucleotides, influence translation or trigger mRNA degradation [10]. In humans, over 3000 miRNAs are implicated in essential cellular processes such as development, differentiation, and apoptosis [11]. Dysregulation of these molecules has been linked to various diseases, including cancer, CHD, DM, and inflammatory conditions like multiple sclerosis and systemic lupus erythematosus [12, 13]. Their dysregulation is also associated with immune response abnormalities and the development of chronic inflammatory diseases, such as periodontitis [14].

In drug research and diagnostics, biomarkers have become invaluable, with miRNAs emerging as particularly promising due to their remarkable stability in diverse biofluids, including blood, gingival crevicular fluid (GCF), saliva, urine, and cerebrospinal fluid [15]. Their release into circulation and stability underscores their potential as non-invasive biomarkers for diagnosing various diseases, including periodontitis [16, 17]. Although conventional clinical diagnosis of periodontitis using parameters such as periodontal probing depth (PPD), clinical attachment loss (CAL), and radiographs is well established, these methods primarily detect disease after significant tissue damage has occurred and may not reliably reflect current disease activity or predict future progression [18, 19]. MicroRNAs, due to their regulatory role in inflammation and immune response, as well as their detectability in minimally invasive samples, are increasingly being explored as adjunctive biomarkers [20]. Their dynamic expression in response to periodontal inflammation supports their potential application in enhancing diagnostic precision and enabling real-time monitoring within periodontal care [21]. The integration of miRNA biomarkers into clinical practice should be considered a complementary approach to conventional diagnostic methods, offering the potential to enhance early detection, refine risk assessment, and facilitate precision medicine in periodontitis management [22]. These miRNA profiles show potential as diagnostic and monitoring biomarkers for periodontitis, which may contribute to earlier detection and more timely interventions, thereby improving patient outcomes [23].

The identification of miRNA as diagnostic biomarkers for periodontitis remains challenging due to methodological variations in sample processing, miRNA detection techniques, disease heterogeneity, and study limitations such as small sample sizes and potential biases in control groups. Additionally, distinguishing periodontitis-specific miRNAs from those influenced by systemic inflammation presents a significant challenge. Despite these complexities, extensive efforts have been made to identify miRNA signatures capable of differentiating periodontitis patients from healthy individuals. While scientific interest in miRNAs related to periodontal health continues to grow, previous reviews have explored this topic from various perspectives, including pathophysiological mechanisms and clinical perspectives [21, 23, 24]. A previous meta-analysis (2020) assessed heterogeneity and publication bias but focussed mainly on differential expression (fold change) rather than diagnostic accuracy [25]. This study aims to address this gap by systematically assessing miRNAs consistently associated with periodontitis. By conducting a meta-analysis and integrating findings on diagnostic accuracy, this analysis evaluates the potential of these miRNAs as biomarkers for early detection of periodontitis.

Methods.

## Review question

Which microRNAs are consistently associated with periodontitis, and what is their diagnostic accuracy based on available evidence?

### Literature search strategy

The present systematic review adhered to the Preferred Reporting Items for Systematic Evaluation and Meta-Analysis (PRISMA) statement [26]. The protocol was registered in PROSPERO under ID: CRD42024520884. Electronic searches were conducted utilising the following databases: Web of Science, Wiley Online Library, PubMed, and Scopus up to 31st May 2025, focussing on publications investigating the association between periodontitis and miRNA. Detailed search strategies were tailored to each database, utilising specific keywords and Boolean connectors (AND, OR), encompassing the following keywords: ([periodontal disease OR periodontic OR periodontitis] AND [microRNA OR miRNA OR miRNA’s] AND [diagnosis OR biomarker OR marker]) with no restriction on the year of publication. For detailed search term strategies, see Appendix 2 in the supplementary files.

### Inclusion and exclusion criteria

This systematic review included studies that explicitly investigated the association between miRNA expression and periodontitis, focussing on their potential as diagnostic biomarkers. Only full-length articles in the English language were included. Studies were eligible if they assessed miRNA expression patterns in periodontitis patients compared to healthy controls, with preference for those applying standardised case definitions, following the 2018 AAP/EFP classification of periodontitis, to ensure comparability across studies. Studies with ≥ 10 patients per group (case and control) were included to account for inter-individual variations and minimise false-positive or false-negative findings.

Studies were excluded if they were clinical case reports, non-human, reviews, meta-analyses, editorials, short communications, or conference abstracts, as these study types do not provide primary data or sufficient methodological detail. Additionally, studies were excluded if they lacked a healthy control group, did not specify diagnostic criteria for periodontitis, or failed to describe clear miRNA detection and validation methodologies to ensure methodological rigour.

The meta-analysis was limited to studies reporting sufficient data to calculate diagnostic accuracy parameters for miRNAs associated with periodontitis, and that reported common miRNAs in at least three independent investigations. Studies that focussed solely on the mechanistic roles of miRNAs or their associations with systemic diseases, without assessing diagnostic performance for periodontitis, were excluded from the meta-analysis but included in the qualitative synthesis if relevant.

To systematically define the study selection criteria for human observational studies evaluating diagnostic accuracy, we applied the PICOTS framework (Population, Intervention, Comparison, Outcome, Timing, Study Design), as outlined in Table 1.

**Table 1:** **PICOTS—population, intervention, comparison, outcomes, timing**, **and study design**

Component	Definition in this study
P (Population)	Patients diagnosed with periodontitis (2018 AAP/EFP classification) and healthy controls
I (Intervention)	Analysis of miRNA expression associated with periodontitis
C (Comparison)	Standard clinical or radiographic diagnostic criteria for periodontitis (CAL, PPD, radiographic bone loss)
O (Outcome)	Diagnostic accuracy of miRNAs: sensitivity, specificity and area under the curve (AUC)
T (Timing)	Time of miRNA sample collection relative to disease status or clinical diagnosis (if reported)
S (Study Design)	Observational human studies (cross-sectional, case–control, cohort) with ≥ 10 participants per group

### Screening of the studies

Two reviewers, MAM and WNS, assessed the titles and abstracts and thoroughly examined the full texts of the chosen articles based on the predetermined inclusion criteria. Discrepancies between the two reviewers were resolved through consensus. In cases of uncertainty or disagreement, a third researcher (MN) was consulted for discussion to achieve consensus. Duplicate articles were removed from consideration, and rationales for article rejections were also recorded.

### Data extraction and study characteristics

The following details were extracted for each study: study methodology, participant groups, type of sample, sample size, mean age, miRNAs analysed, and main outcomes.

Two researchers (MAM and WNZ) independently performed data extraction. A third researcher (MN) was consulted in cases of uncertainty. After completing the extraction, the reviewers cross-checked their findings to ensure consistency and accuracy. The involvement of a third reviewer (MN) helped mitigate biases and ensured a thorough assessment of the extracted data. Through this systematic and collaborative approach to data extraction, the review aimed to minimise errors and enhance the reliability of the synthesised evidence.

### Qualitative assessment

The methodological quality and risk of bias of the included diagnostic studies were assessed using the QUADAS-2 tool, which evaluates four key domains: patient selection, index test, reference standard, and flow and timing [27]. Each domain is judged for risk of bias, and the first three domains are also assessed for concerns regarding applicability. Studies were classified as having low, high, or unclear risk of bias in each domain. Two independent reviewers (MAM and WNZ) performed the assessments, with a third reviewer (MN) consulted to resolve any disagreements.

### Meta-analysis

A meta-analysis was conducted to evaluate the pooled sensitivity and specificity of miRNA biomarkers, enabling meaningful statistical pooling. True positive (TP), false positive (FP), false negative (FN), and true negative (TN) values were extracted from each study to estimate diagnostic accuracy. The pooled estimates of each miRNA were visualised using forest plots summarising sensitivity and specificity estimates reported in each study, while hierarchical summary receiver operating characteristic (HSROC) curves were generated to illustrate the trade-off between sensitivity and specificity across included studies. These curves incorporated the pooled summary point. Individual study points were labelled, and in the combined HSROC visualisation, data points were colour-coded according to miRNA type. For each miRNA, AUC values were estimated using the bivariate random-effects Reitsma model.

For each miRNA, a bivariate random-effects model with Restricted Maximum Likelihood (REML) estimation was applied to compute pooled sensitivity and specificity. This model jointly estimates between-study variances for logit sensitivity and logit false-positive rate, as well as their covariance, thereby accounting for the correlation between these two diagnostic accuracy parameters. A heterogeneity test was performed before model selection. Based on observed between-study heterogeneity, with I^2^ values exceeding 50% in all comparisons, a random-effects model was justified. Heterogeneity was assessed using Cochran’s Q test, I^2^ statistic, tau squared (τ^2^), and associated p-values. Where data allowed, analyses were stratified by specimen matrix and assay platform using prespecified moderators to explore potential sources of variability.

Publication bias was assessed using Deeks’ funnel plot asymmetry test, where applicable. Sensitivity analyses and leave-one-out diagnostics were performed to assess the impact of individual studies on pooled estimates and heterogeneity metrics, applying an a priori outlier policy (studies were flagged as influential if sensitivity or specificity equaled 1.00, < 0.50, or if total misclassification was disproportionately high/low).

All statistical analyses were conducted in R (version 2025.05.1) using the meta and metafor packages, with HSROC curves plotted using custom R functions (add_hsroc()) for each miRNA. The rma() function from metafor was used for random-effects modelling and for estimating heterogeneity statistics (I^2^, τ^2^, H^2^). Visual outputs, including forest plots, were produced using Review Manager (RevMan 5.4, Cochrane Collaboration).

### Certainty of evidence assessment

The certainty of evidence for diagnostic accuracy outcomes was assessed using the GRADE approach for diagnostic test accuracy (GRADE-DTA). Evidence was evaluated across five domains: risk of bias, inconsistency, indirectness, imprecision, and publication bias. Risk of bias was informed by QUADAS-2 assessments, while inconsistency and imprecision were judged based on between-study heterogeneity and the width of confidence intervals. Publication bias was assessed using Deeks’ funnel plot asymmetry test when sufficient studies were available. The overall certainty of evidence for each miRNA biomarker was rated as high, moderate, low, or very low.

## Results

### Study selection

The search strategy was conducted across four different databases, resulting in a total of 552 documents: 246 from PubMed, 90 from Web of Science, 128 from Scopus, and 88 from Wiley Online Library. After removing 158 duplicates, 394 unique articles remained for title and abstract screening. No additional articles were identified by manual searching. During the initial screening of titles and abstracts, 219 articles were excluded due to irrelevance. Of the remaining 175 articles, an additional 143 articles were excluded for the following reasons: literature reviews (n = 67), editorials or short communications (n = 1); pilot studies (n = 11); studies unrelated to the research question or focussed on other pathologies and conditions (n = 41); retracted studies (n = 3); correction notices (n = 1); and full-text unavailability (n = 22).

**Fig. 1:**
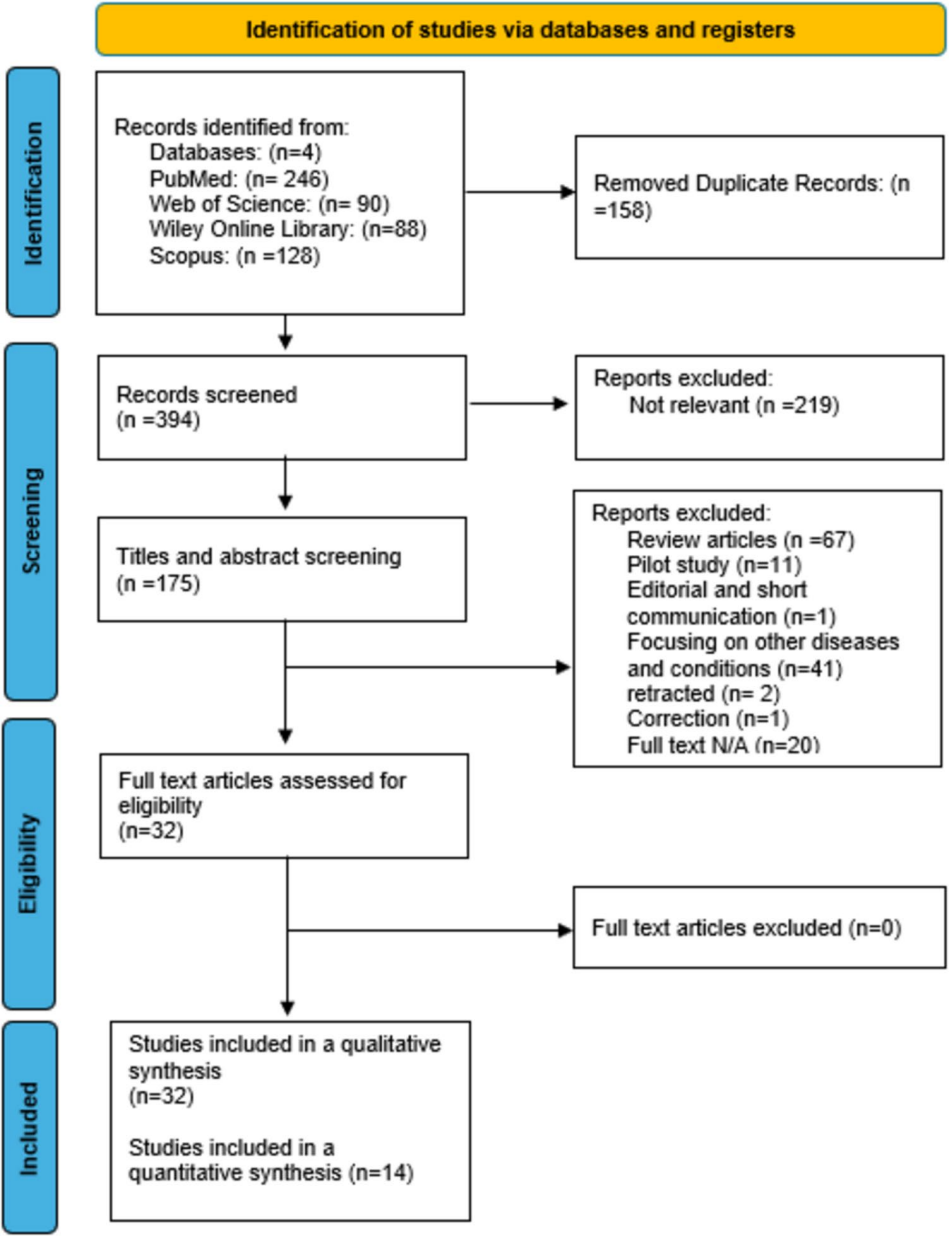
Flow diagram of the literature selection process based on PRISMA

Following this, 32 articles fulfilled all the inclusion and exclusion criteria and were included in this systematic review; 14 studies were included in a quantitative synthesis (meta-analysis). The PRISMA flow diagram (Fig. 1) summarises the article selection process.

### Characteristics of included studies

The characteristics of the included studies are summarised in Table 2. A total of 32 articles were included, all of which examined miRNA expression in periodontitis. These studies comprised 21 case–control, 1 prospective cohort, 1 retrospective case–control, and 9 cross-sectional studies. Sample sizes ranged from 22 to 230 participants, with an age range of 28 to 75 years. All studies compared miRNA expression in individuals with periodontitis and healthy controls. Of these, 20 studies involved healthy and periodontitis subjects, 6 included individuals with DM, and 2 involved CHD patients. The analysis of miRNA expression was conducted on various sample types. The most used sample was GCF, followed by saliva, periodontal gingival tissue, blood, serum, and sub-gingival plaque. Notably, five studies utilised multiple sample types: two studies analysed gingival tissue, GCF, saliva, and blood plasma, two studies analysed both GCF and serum, and one study analysed blood, saliva, and GCF. All studies assessed broad and specific miRNA expression profiles using various techniques, including reverse transcription polymerase chain reaction (qRT-PCR), microarray, and next-generation sequencing.

**Table 2:** Characteristics of included human studies

**Study type**	**Type of sample**	**Participant groups**	**Sample size**	**Mean age** **(years)**	**Analysed and/or deregulated miRNAs**	**Main outcomes**	**References**
Case–Control	GCF	G1: CP G2: HP	22 G1: 11 G2: 11	G1: 34.4 G2: 43.95	miR-30a-5p, miR-199b-3p, miR-338-5p, and miR146a-5p	miR-199b-3p and miR-146-5p were positively correlated with GCF volume (p < 0.004; p < 0.027, respectively), supporting their potential as chronic periodontitis biomarkers	[28]
Case–Control	GCF	G1: CP G2: HP	169 G1: 91 G2: 78	G1: 40.96 ± 10.58 G2: 40.86 ± 9.58	miR-199a-3p	miR-199a-3p shows potential as a biomarker for periodontitis (p < 0.05)	[29]
Case–Control	Saliva and GCF	G1: CP G2: HP	240 G1: 150 G2: 90	> 18	miR-181 family	Expression levels of miR-181 family members in oral biofluids were significantly associated with periodontitis severity (p < 0.05) and demonstrated potential as diagnostic biomarkers for periodontitis	[30]
Case–Control	GCF	G1: CP G2: HP	42 G1: 28 G2: 14	20–60	miR-223 miR-214	miR-214 is abundant in healthy tissue (p < 0.05), while miR-223 is elevated in periodontitis, especially in smokers. miR-214 shows the highest diagnostic accuracy (p < 0.05)	[31]
Case–Control	Gingival tissue	G1: CP G2: HP	50 G1: 17 G2: 33	45.3	miR-29b-3p, miR-34a-5p, miR-155-5p, miR-181a-5p, and miR-192-5p	miR-29b-3p, miR-34a-5p, miR-155-5p, miR-181a-5p, and miR-192 are upregulated in periodontitis, indicating their role in disease pathogenesis and potential as early diagnostic biomarkers (p < 0.05)	[32]
Case–Control	Gingival tissue	G1: CP G2: HP G3: T2DM G4: CP + T2DM	48 G1: 12 G2: 12 G3: 12 G4: 12	N/S	miR-155	miR-155 could be a potential diagnostic biomarker for diabetes-associated PD (p < 0.05)	[33]
Case–control	Saliva	G1: HP G2: Gingivitis G3: CP G4: CP + T2DM	52 G1: 13 G2: 13 G3: 13 G4: 13	> 18	miR-21	miR-21 may serve as a diagnostic marker for periodontitis severity (p < 0.05)	[34]
Case–Control	Saliva	G1: CP G2: HP	80 G1: 40 G2: 40	23–56	miR-146a	miR-146a is elevated in periodontitis and may serve as a diagnostic and prognostic marker (p < 0.05)	[35]
Case–control	GCF	G1: HP G2: Patients with Stage III periodontitis	100 G1: 50 G2: 50	G1: 34.4 G2: 43.95	miR-223	miR-223 and RAB12 show potential as biomarkers for periodontitis (p < 0.01)	[36]
Case–control	Unstimulated Saliva	G1: HP G2: CP	50 G1: 25 G2: 25	G1: 35.78 G2: 49.62	miR-221-5p, miR-222-5p, miR-223-5p	Elevated levels of miR-221-5p, 222-5p, and 223-5p in saliva may serve as biomarkers for diagnosing and predicting periodontitis (p < 0.01)	[37]
Case control	Unstimulated saliva Blood, saliva, GCF	G1: HP G2: CP	100 G1: 50 G2: 50	G1: 31.15 G2: 47.66	miR-223-5p	Elevated miR-223-5p levels in plasma, saliva, and GCF can serve as a potential diagnostic biomarker for periodontal disease (p = 0.05)	[38]
Case–control	Whole blood samples	G1: control group G2: generalised periodontitis G3: CHD with healthy periodontium atherosclerosis with a clinically healthy periodontium G4: CHD with generalized periodontitis	120 G1: 30 G2: 30 G3: 30 G4: 30	35–75	miR-155	miR-155 levels may serve as an inflammatory biomarker for the diagnosis and prediction of periodontitis and CHD severity (p < 0.001)	[39]
Case control	Blood samples	G1: CP G2: HP	146 G1: 86 G2: 60	N/S	miR-205	miR-205 and HMGB1 are linked to periodontitis progression (p < 0.05); miR-205 may modulate inflammatory cytokines (IL-1β, IL-6, TNF-α) and serve as a potential biomarker for periodontitis	[40]
Case–control	Gingival tissue	G1: CP G2: HP	30 G1: 20 G2: 10	28–63	miR-146a	miR-146a levels were significantly higher in patients and positively correlated with clinical parameters (p < 0.05). This elevation was associated with a significant reduction in TNF-α and IL-6 levels (p < 0.001)	[41]
Case–control	Gingival tissue	G1: systemically healthy patients with localised stage III/IV periodontitis G2: systemically and periodontally healthy subjects	98 G1: 49 G2: 49	N/S	miR-155	miR-155 is upregulated in periodontitis and may serve as a biomarker for distinguishing periodontal health from disease (p < 0.001)	[42]
Case–control	Serum	G1: CP G2: HP	60 G1: 30 G2: 30	N/S	miR-664a-3p, miR-501-5p, miR-21-3p	Increased serum levels of miR-664a-3p, miR-501-5p, and miR-21-3p in periodontitis patients suggest these miRNAs as potential biomarkers for chronic periodontitis (p < 0.05)	[43]
Case–control	GCF	G1: CP G2: HP	36 G1: 21 G2: 15	56–63	miR-23a-3p, miR-423-5p, miR-15a-5p, miR-223-3p, miR-103a-3p	Upregulation of miR-103a-3p (p < 0.023), miR-23a-3p (p < 0.001), miR-15a-5p (p < 0.01), miR-223-3p (p < 0.001) in periodontitis; TNFα and IL-6 levels linked to disease severity (p < 0.001)	[44]
Case–control	GCF	G1: CP G2: HP	216 G1: 103 G2: 113	N/S	miR-200a-3p, miR-200a-5p, miR-200b-3p, miR-200b-5p, miR-200c-3p, miR-200c-5p	MiR-200a, -200b, and -200c in GCF are potential diagnostic biomarkers and therapeutic targets for CP (p < 0.05)	[45]
Case–control	GCF	G1: CP G2: HP	180 G1:80 G2: 100	N/S	miR-30b-3p, miR-125b-1-3p	Overexpression of miR-30b-3p and miR-125b-1-3p has been linked to the development and progression of periodontitis (p < 0.05)	[46]
Case–control	Gingival tissue	G1: CP G2: HP	57 G1: 29 G2: 28	N/S	miR-191-3p, miR-221-3p, miR-224-5p, miR-1228-3p	Altered miRNA expression in gingival tissue, with miRNAs related to angiogenesis and EMT serving as potential diagnostic or prognostic biomarkers for periodontal disease	[47]
Retrospective case–control	GCF	G1: CP G2: HP	147 G1: 76 G2: 71	GI: 47 G2: 48	miR-28-5p	miR-28-5p is a potential diagnostic biomarker for CP and may impact disease progression by targeting SPHK1 (*p* < 0.05)	[48]
Prospective cohort	Saliva	G1: T2DM only G2: T2DM/OP + PD) G3:T2DM/OP G4: healthy individuals	187 G1: 45 G2: 40 G3: 50 G4: 52	Less than 60	miR-25-3p	miR-25-3p expression may be induced by T2DM and increased with progression to coexistent osteoporosis and PD (p < 0.05)	[49]
Cross-sectional study	GCF	G1: CP G2: HP	70 G1: 18 G2: 52	N/S	miR-1226	miR-1226 may serve as a diagnostic marker for periodontitis and indicate its severity	[50]
Cross-sectional study	Gingival tissue, saliva, blood plasma	G1: CP G2: HP	230 G1: 144 G2: 86	N/S	miR-140-3p, miR-145-5p, miR-146a-5p, miR-195-5p	Gingival miR-140-3p (*p* = 0.013), -145-5p (p ≤ 0.001), and -125a-3p (p = 0.001) were independently associated with PD presence and severity; Salivary and plasma miRNA levels were variably related to PD	[51]
Cross-sectional	GCF and serum	G1: CP with systemically healthy G2: type 2 diabetic chronic periodontitis group G3: control group systemically healthy, no history of periodontitis	60 G1: 20 G2: 20 G3: 20	N/S	miR-223, miR-203, miR-200b	miR-223 (p = 0.00031), miR-203 (p > 0.05), and miR-200b (p = 0.046) have distinct expression profiles in chronic periodontitis with and without type 2 diabetes, with miR-223 showing potential as a serum biomarker and involvement in disease pathogenesis	[52]
Cross-sectional	GCF, Serum	G1: periodontitis without T2DM G2: T2DM G3: periodontitis + T2DM G4: Healthy	97 G1: 26 G2: 24 G3: 22 G4: 25	N/S	miR-223, miR-203, miR-200b	Differential expressions of miR-223 and miR-200b in periodontal-diseased patients with and without T2DM. Both miRNAs are correlated with periodontal disease pathogenesis and T2DM susceptibility (p < 0.05)	[53]
Case–control	GCF	G1: CP G2: CP + DMT2 G3: HP G4: healthy periodontium with T2DM	96 G1: 24 G2: 24 G3: 24 G4: 24	44.13 ± 12.46	miR-146a, miR-155	miR-146a and miR-155 may be considered as possible novel biomarkers for periodontitis in nondiabetic and type 2 diabetic patients (p < 0.01)	[54]
Cross-sectional	Sub-gingival plaque samples	G1: CP + CHD G2: CP G3: HP	90 G1: 30 G2: 30 G3: 30	35–36	miR-146a	miRNA‐146a is involved in the pathogenesis of both periodontitis and coronary heart disease (p < 0.01)	[55]
Cross-sectional	Gingival tissue, GCF, saliva, blood and plasma	G1: CP G2: HP	61 G1: 30 G2: 31	N/S	miR-199a-5p, miR-483-5p, miR-3198, miR-4299	miR-199a-5p, miR-483-5p, miR-3198, and miR-4299 are associated with PD (p < 0.05). Specific miRNAs in GCF and plasma show diagnostic potential	[56]
Cross-sectional	GCF	G1: CP G2: periodontally healthy individuals with PD (with or without RA)	210 G1: 134 G2: 76	N/S	miR-140-3p, miR-145-5p, miR-146a-5p, miR-195-5p	miR-146a-5p levels in GCF negatively correlated with periodontitis severity s (p < 0.05). MiR-140-3p and miR-145-5p levels were higher in severe PD (p < 0.05). Combined, these miRNAs showed strong diagnostic potential (p < 0.05)	[57]
Cross-sectional	Saliva	G1: CP G2: HP	76 G1: 41 G2: 35	G1: 29.78 G2: 28.59	miR-155, miR-146a	MiR-155 and miR-146a were highly expressed in PD patients; their levels correlated with PD severity and clinical indexes (p < 0.05), suggesting their involvement in PD progression	[58]
Cross-sectional	Saliva	G1: CP G2: HP	70 G1: 35 G2: 35	G1: 31–67 G2: 32–69	miR-1246	Elevated miR-1246 levels in saliva were associated with periodontal indices, inflammatory cytokines, and protease molecules, suggesting their involvement in chronic periodontitis development (p < 0.05)	[59]

*CP* chronic periodontitis, *HP* healthy participants, *miR* microRNA, *G*: Group; *GCF* gingival crevicular fluid, *RAB12* Ras-related protein Rab-12, *CHD* coronary heart disease; PD: periodontitis; RA: rheumatoid arthritis, *HMGB1* high-mobility group box 1, *IL-1β*: interleukin-1 beta, *IL-6* interleukin-6; *TNF-α* tumor necrosis factor-alpha, *EMT* epithelial–mesenchymal transition, *SPHK* sphingosine kinase; *PD* periodontal disease, *T2DM* type 2 diabetes mellitus, *N/S* not specified

### Quality assessment of selected studies

**Fig. 2:**
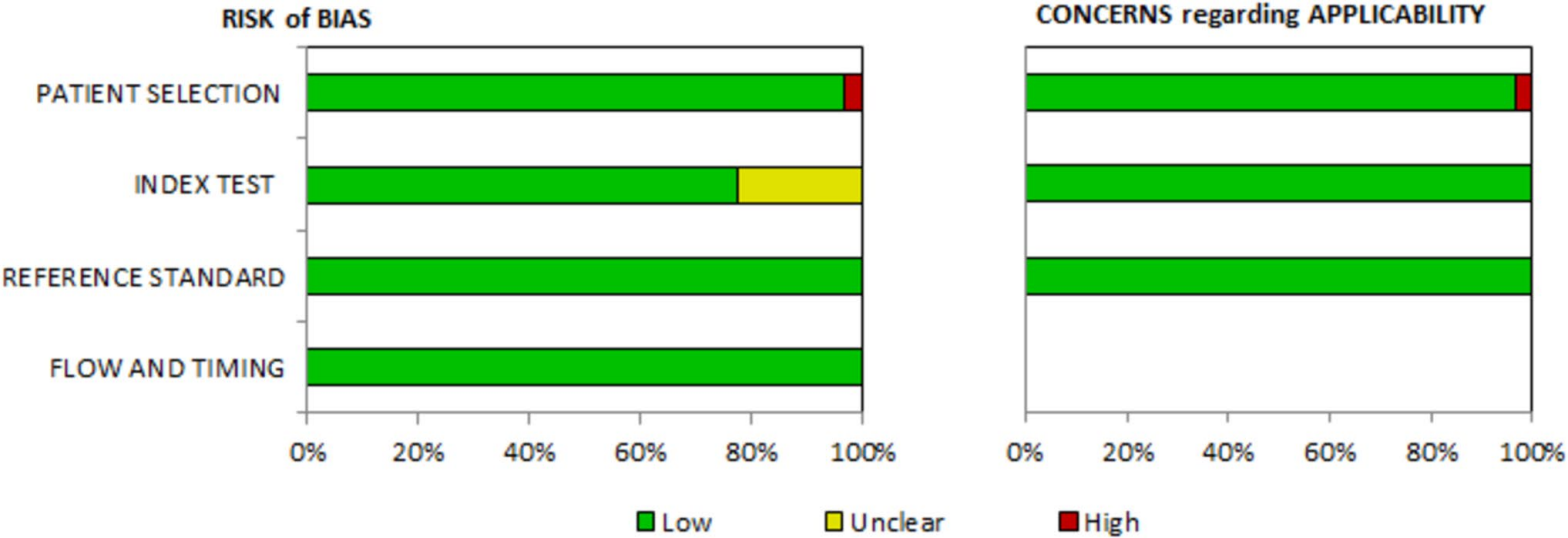
Risk of bias and applicability assessment summary of included studies using QUADAS-2. The green region represents studies with a low risk of bias, yellow indicates a moderate risk, and red indicates a high risk

We qualitatively assessed the included studies using the QUADAS-2 tool, as detailed in Table S1. Figure 2 presents the overall risk of bias across all studies. Most studies clearly defined patient selection criteria, used well-established definitions of periodontal disease, and applied appropriate index tests and reference standards. Overall, the majority of studies were judged to be at low risk of bias and had low concerns regarding applicability, indicating high methodological quality. Out of the 31 studies, 7 showed an unclear risk of bias for the index test [51–56, 58], and one study showed a high risk of bias in patient selection due to the inclusion of only male subjects and multiple exclusions after screening [55].

### Diagnostic accuracy

A total of 14 studies comprising 1,177 participants were included in the diagnostic test accuracy meta-analysis, evaluating three commonly reported microRNA biomarkers: miR-146 (3 studies), miR-155 (5 studies), and miR-223 (6 studies). Pooled sensitivity and specificity estimates were obtained using a bivariate random-effects (Reitsma) model, with heterogeneity, stratified analyses, influence diagnostics, predictive values, and publication bias assessed according to prespecified criteria.

### Overall diagnostic performance

**Fig. 3:**
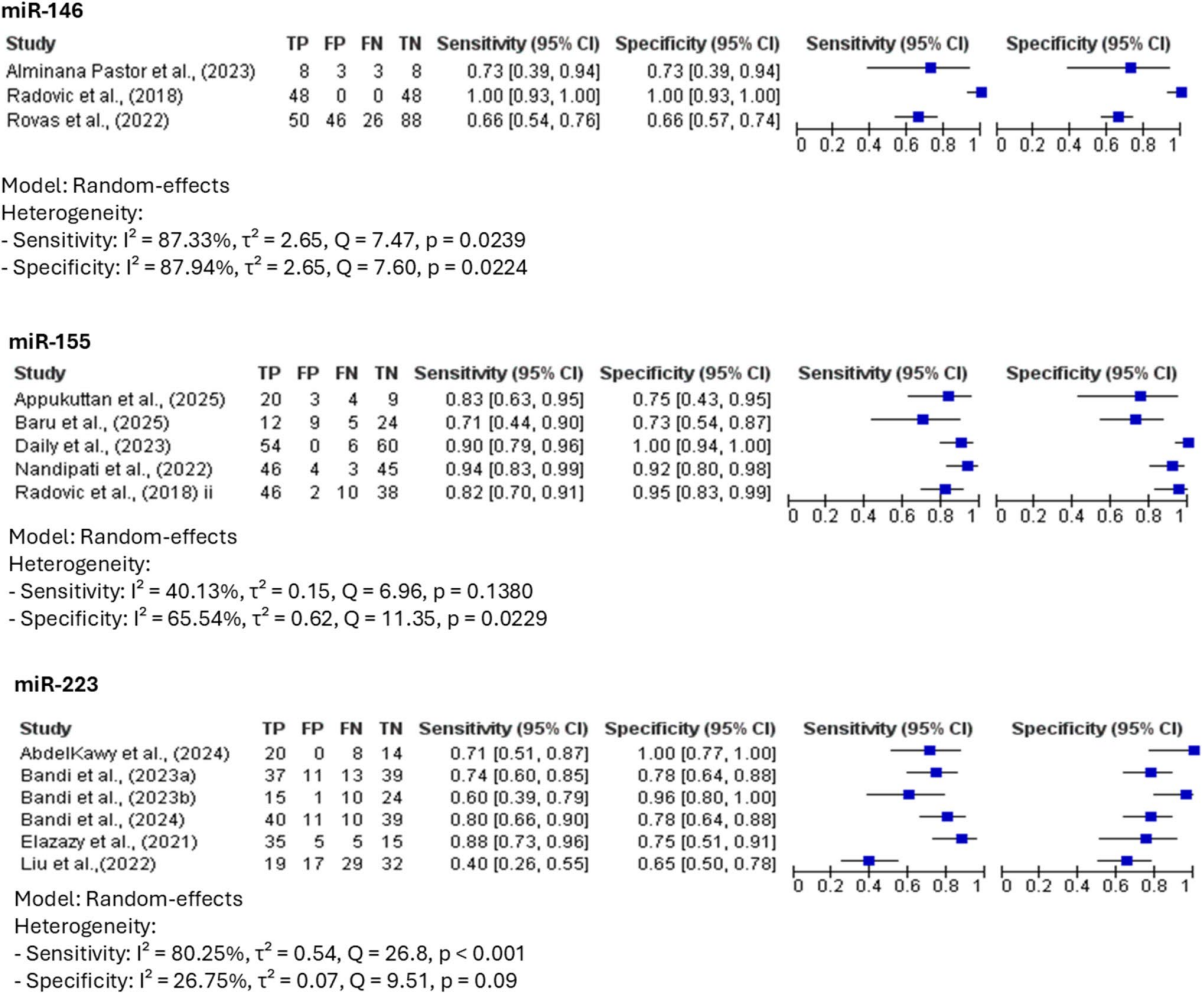
Forest plots of pooled sensitivity and specificity

The overall pooled diagnostic performance for miR-146 demonstrated high accuracy, with pooled sensitivity ≈ 0.87, specificity ≈ 0.87, and HSROC AUC ≈ 0.93. Substantial heterogeneity was observed for both sensitivity and specificity (I^2^ ≈ 87–88%). For miR-155, pooled sensitivity ≈ 0.84, specificity ≈ 0.88, and HSROC AUC ≈ 0.90, with moderate heterogeneity, particularly for specificity. miR-223 showed moderate diagnostic performance with pooled sensitivity ≈ 0.70, specificity ≈ 0.77, and HSROC AUC ≈ 0.79. Considerable heterogeneity was observed for sensitivity (I^2^ ≈ 80%), whereas specificity heterogeneity was lower. These findings are summarised in the forest plots (Fig. 3) and Table 3.

**Table 3:** Diagnostic accuracy parameters for evaluated miRNA biomarkers

Parameter	miR-146	miR-155	miR-223
No. of studies (k)	3	5	6
Pooled sensitivity	0.84 (0.60–0.93)	0.85 (0.77–0.91)	0.70 (0.55–0.84)
Pooled specificity	0.84 (0.60–0.93)	0.84 (0.69–0.93)	0.77 (0.68–0.85)
HSROC AUC	0.928	0.903	0.787
Sensitivity heterogeneity (I^2^)	87.3%	40.1%	80.0%
Specificity heterogeneity (I^2^)	87.9%	65.5%	27.0%,
Influential studies (a priori)	1	2	1
Publication bias (Deeks’)	Not assessed	No evidence	Borderline (p = 0.0544)

### HSROC analysis

**Fig. 4:**
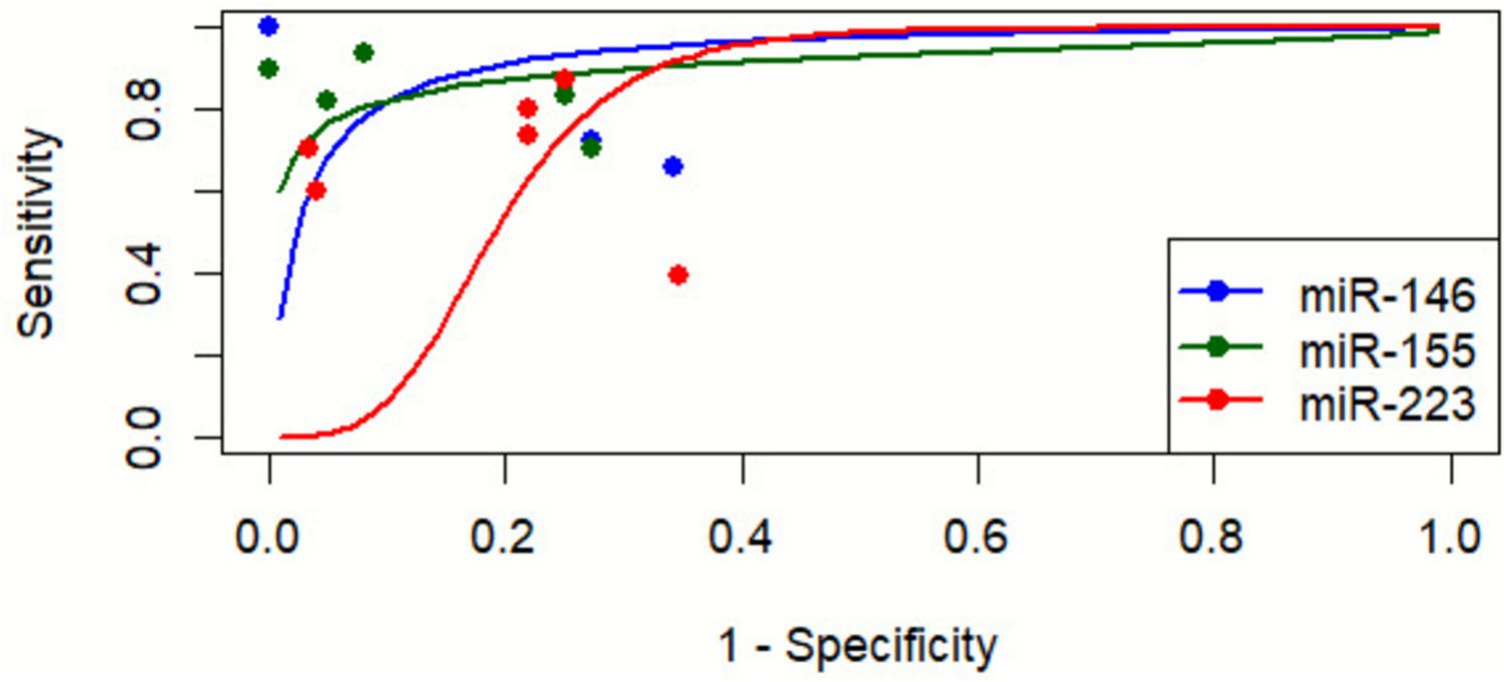
HSROC curve

The HSROC plot (Fig. 4) demonstrates that miR-146 (blue circles) and miR-155 (green circles) cluster toward the upper-left region of the curve, indicating high diagnostic accuracy. In contrast, miR-223 (red circles) shows wider dispersion across the plot, reflecting greater between-study heterogeneity and variability related to specimen types. Separate HSROC curves with 95% confidence regions for each miRNA are provided in the Supplementary material.

### Between-study variance–covariance

The bivariate random-effects models estimated the between-study covariance between logit sensitivity and logit false-positive rate for each miRNA. A negative covariance was observed for all three miRNAs, indicating an inverse relationship between sensitivity and false-positive rate across studies. The magnitude of covariance was largest for miR-146, consistent with substantial heterogeneity and potential threshold effects, whereas miR-155 showed a smaller covariance, reflecting greater stability of diagnostic performance. miR-223 demonstrated a weak negative covariance, suggesting that heterogeneity was driven predominantly by factors other than threshold variation. Full variance–covariance matrices are provided in Supplementary Table S2.

### Stratified analyses by specimen matrix and assay platform

Where data allowed, analyses were stratified by specimen matrix (serum, saliva, plasma) and assay platform (qPCR). miR-146 showed consistent accuracy across serum and saliva, with a pooled sensitivity of 0.85–0.87 and a specificity of 0.83–0.87. miR-155 performed slightly better in plasma than serum, with minimal variation in pooled specificity. miR-223 exhibited higher variability between serum and saliva, reflecting heterogeneity in study populations. All studies included in the analysis used qPCR; no alternative assays were reported. Full stratified results are presented in Table 4.

**Table 4:** Stratified pooled sensitivity and specificity of miRNAs by specimen matrix and assay platform

miRNA	Stratification	No. of studies	Pooled sensitivity	Pooled specificity	Notes
**miR-146**	Serum	2	0.85	0.83	Moderate heterogeneity
	Saliva	1	0.87	0.87	Single study
	qPCR	3	0.86	0.86	All studies used qPCR
	Other assay	0	–	–	Not reported
**miR-155**	Serum	3	0.83	0.88	Moderate heterogeneity
	Plasma	2	0.85	0.87	Slight variation in specificity
	qPCR	5	0.84	0.88	All studies used qPCR
	Other assay	0	–	–	Not reported
**miR-223**	Serum	3	0.68	0.75	High heterogeneity
	Saliva	3	0.72	0.79	Moderate heterogeneity
	qPCR	6	0.70	0.77	All studies used qPCR
	Other assay	0	–	–	Not reported

Pooled estimates were calculated using the bivariate random-effects Reitsma model. Some strata contain only one study; heterogeneity metrics are therefore not meaningful.

### Influence diagnostics

Influence diagnostics using the prespecified a priori outlier policy identified one study for miR-146, two studies for miR-155, and one clearly influential study for miR-223, with several additional studies showing moderate influence. Leave-one-out analyses confirmed the sensitivity of pooled estimates to these studies for miR-146 and miR-155, while pooled estimates for miR-223 remained robust. Full influence diagnostics and leave-one-out results are provided in the Supplementary Table S3.

### Predictive values (PPV and NPV)

PPV and NPV were calculated for each miRNA across plausible disease prevalence scenarios, reflecting low (10%), moderate (30%), and high (50%) periodontal disease prevalence in typical populations. Calculations incorporated uncertainty around pooled sensitivity and specificity estimates using the delta method to estimate confidence intervals. miR-155 consistently demonstrated the most favourable balance of predictive values across all prevalence scenarios. miR-146 showed strong predictive performance, though PPV and NPV varied slightly by sample type. miR-223 exhibited lower PPV at low prevalence, reflecting its reduced sensitivity, while NPV remained relatively high even in low-prevalence scenarios. Full estimates with uncertainty intervals are provided in Supplementary Table S4.

### Publication bias

Publication bias was not assessed for miR-146 due to the limited number of studies (k = 3). Deeks’ funnel plot asymmetry test showed no significant publication bias for miR-155, whereas borderline evidence was observed for miR-223 (p ≈ 0.054).

### Certainty of evidence

According to the GRADE-DTA framework, the certainty of evidence varied across miRNA biomarkers. For miR-146, the certainty of evidence was rated as low due to serious inconsistency and imprecision, despite a low risk of bias and direct evidence. For miR-155, the certainty of evidence was rated as moderate, with a single downgrade for inconsistency related to between-study heterogeneity. For miR-223, the certainty of evidence was rated as low owing to substantial heterogeneity and concerns regarding publication bias. No serious limitations were identified for risk of bias or indirectness for any of the evaluated biomarkers. Comprehensive GRADE evidence profiles documenting these assessments appear in Supplementary Table S5.

## Discussion

Early and accurate diagnosis plays a crucial role in improving disease management and guiding effective therapeutic strategies. With ongoing advances in miRNA research, periodic reassessment of their diagnostic potential remains essential to validate clinical utility. This systematic review provides current evidence from studies assessing miRNAs consistently associated with periodontitis. The meta-analysis demonstrated that miR-146 and miR-155 exhibit strong diagnostic potential (HSROC AUC ≈ 0.93 and 0.90, respectively), while miR-223 showed moderate accuracy (AUC = 0.78) with greater between-study variability.

Among the analysed miRNAs, miR-146 demonstrated the highest diagnostic accuracy, with pooled sensitivity and specificity of ~ 0.87 each. Despite this promising performance, substantial heterogeneity was observed for miR-146 and miR-223, while miR-155 exhibited moderate heterogeneity. Influence diagnostics and leave-one-out analysis identified one to two studies with disproportionate impact on pooled estimates, supporting the robustness of pooled results.

Functionally, miR-146 is strongly associated with periodontitis [28, 35, 41, 51, 54, 55, 57, 58] and may regulate pro-inflammatory cytokines via a negative feedback loop [60]. Overexpression of miR-146a has been linked to decreased levels of TNF-α, IL-1β, and IL-6, suggesting additional inflammatory or non-immunologic pathways contribute to disease progression. Its levels positively correlate with CAL and other disease severity measures [35, 41]. Conversely, under-expression of miR-146a in some studies suggests a potential role in prolonged inflammatory conditions such as aggressive periodontitis [57]. Additionally, miR-146a has been associated with systemic comorbidities, including coronary heart disease, indicating its dual role in local and systemic inflammatory modulation [55].

Similarly, miR-155 demonstrated strong diagnostic accuracy (sensitivity ≈ 0.84, specificity ≈ 0.88). Deeks’ test indicated minimal evidence of publication bias, supporting the reliability of pooled estimates. miR-155 is involved in innate immune regulation and inflammatory amplification [32, 33, 39, 42, 58]. Additionally, miR-155 was found to be elevated in the GCF of chronic periodontitis patients and correlates positively with superoxide dismutase activity, indicating its role in the pathogenesis of periodontitis and T2DM (r = 0.64) [54]. Stratified analyses by sample type confirmed consistent diagnostic performance across different biological matrices, reinforcing clinical applicability.

miR-223 showed moderate diagnostic performance (sensitivity ≈ 0.70, specificity ≈ 0.77). miR-223 remains biologically relevant due to its established role in regulating neutrophil function and osteoclast differentiation, key processes in periodontal inflammation and bone resorption [31, 36–38, 44, 52, 53, 61]. Its dysregulation has been linked to several inflammatory disorders, contributing to macrophage activation, osteoclast differentiation, and tissue degradation [62]. As a central regulator of osteoclastogenesis, miR-223 contributes to alveolar bone loss in periodontitis and may affect systemic metabolic conditions such as T2DM [52, 53, 63, 64].

Elevated levels of the miR-200 family in the GCF of chronic periodontitis patients suggest potential diagnostic utility, demonstrating an AUC of 0.997 with 99.03% sensitivity and 98.23% specificity. These miRNAs positively correlated with clinical periodontal parameters [45]. Supporting this, Elazazy et al. (2021) found a significant association between miR-200b, CAL, PPD, and TNF-α levels (p < 0.05), indicating involvement in periodontal inflammation [52]. However, due to the limited number of studies, a quantitative meta-analysis could not be performed for this family of miRNAs.

For a diagnostic tool to be deemed acceptable, an AUC greater than 0.8 is required [65]. Among the various miRNAs suggested as diagnostic biomarkers, those that could be reliably used for diagnosing periodontitis include miR-200c-3p [45], miR-200b [53], miR-200a-5p [45], miR-200a-3p, miR-223-5p [38], miR-125a-3p, miR-140-3p, and miR-145-5p [51], miR-200c-5p, miR-200b-3p [45], miR-29b-3p, miR-34a-5p, miR-181a-5p, and miR-192-5p [32]. These miRNAs proposed by researchers as potential biomarkers, despite having AUC values close to 0.8, do not demonstrate adequate performance for diagnostic use.

This review demonstrates that miRNAs hold promise as non-invasive biomarkers for periodontitis, with potential application in chairside or point-of-care testing. PPV and NPV analyses across plausible prevalence scenarios indicate that miR-146 and miR-155 provide more reliable predictive performance than miR-223, emphasising their potential clinical utility. Such tools could be particularly valuable in low-resource settings, enabling earlier detection and personalised care compared to conventional diagnostic methods. Compared to previous systematic reviews, this study offers a more integrated assessment of diagnostic performance by combining a systematic review with a diagnostic accuracy meta-analysis. Evaluating miR-146, miR-155, and miR-223 in parallel allows direct comparison of diagnostic potential and identification of the most promising candidates. Key methodological strengths include stratified analyses, robust influence diagnostics, leave-one-out testing, and certainty assessment using GRADE-DTA, enhancing the reliability of pooled estimates.

Despite these strengths, several limitations should be acknowledged. First, some included studies had relatively small sample sizes, which may have reduced the precision and stability of pooled diagnostic estimates. Second, substantial residual heterogeneity persisted, likely reflecting differences in study populations, specimen types, detection platforms, and applied cut-off thresholds, despite stratified analyses. The lack of fully standardised protocols for sample collection, handling, and miRNA quantification further limits comparability across studies and may influence diagnostic accuracy. Finally, although most studies adjusted for key confounders, inconsistent control of factors such as smoking, DM, and CHD, which are known to influence miRNA expression, adds further complexity to biomarker interpretation [33, 34, 39, 49, 52–55].

Given the current evidence, the identified miRNAs should be considered preliminary diagnostic candidates requiring further validation before clinical application. Future studies should focus on external validation in large, diverse cohorts and assess whether miRNAs improve diagnostic performance beyond conventional clinical parameters. Longitudinal studies are needed to evaluate predictive value over time, with stratification by disease stage. Integrating multiple miRNAs into a panel may further enhance diagnostic accuracy and clinical utility.

## Conclusions

In conclusion, miR-146 and miR-155 demonstrate strong diagnostic accuracy for periodontitis, whereas miR-223 shows only moderate performance with greater heterogeneity. Despite their promise as non-invasive biomarkers, clinical translation is currently limited by substantial between-study heterogeneity, lack of standardised cut-off values, and absence of external validation. Other miRNAs identified in the systematic review could not be meta-analysed due to insufficient data. Standardisation of sample collection and miRNA quantification protocols is essential for improving comparability and reliability. Future large-scale, longitudinal studies should validate these findings and assess whether miRNA panels can enhance diagnostic performance beyond conventional periodontal assessment.

## Supplementary Information

Below is the link to the electronic supplementary material.

Supplementary file1 (DOCX 87 KB)

## Data Availability

Data is provided within the manuscript and supplementary material files.
