# Supplementary material for: MicroRNAs as diagnostic biomarkers in periodontitis: a systematic review and meta-analysis
Source: Odontology. 2026 Jan 12;114(3):1178–95. doi: 10.1007/s10266-025-01299-8 (PMC13320082; doi:10.1007/s10266-025-01299-8)

**MicroRNAs as Diagnostic Biomarkers in Periodontitis: A Systematic Review and Meta-Analysis**

Maaz Anwer Memon^a^, Wan Nazatul Shima Shahidan^b^, Noriko Mizusawa^c^, Thirumulu Ponnuraj Kannan^a,d^, Rizwan Mahmood^a^, Khairul Mohd Fadzli Mustaffa^e^, Usman Ashraf^a^

^a^ School of Dental Sciences, Universiti Sains Malaysia, Health Campus, 16150, Kubang Kerian, Kelantan, Malaysia.

^b^ Department of Pharmacology, School of Medical Sciences, Universiti Sains Malaysia, Health Campus, 16150, Kubang Kerian, Kelantan, Malaysia

^c^ Department of Oral Bioscience, Institute of Biomedical Sciences, Tokushima University Graduate School, 3-Kuramoto-cho, Tokushima City 7708504, Japan.

^d^ Human Genome Centre, School of Medical Sciences, Universiti Sains Malaysia, Health Campus, 16150, Kubang Kerian, Kelantan, Malaysia

^e^ Institute for Research in Molecular Medicine (INFORMM), Universiti Sains Malaysia, Health Campus, 16150, Kubang Kerian, Kelantan, Malaysia.

**Corresponding author:** Wan Nazatul Shima Shahidan

**Contact number:** +60125709956

**Address:** Department of Pharmacology, School of Medical Sciences, Universiti Sains Malaysia, Health Campus, 16150, Kubang Kerian, Kelantan, Malaysia.

**Email:** [shima@usm.my](mailto:shima@usm.my)

**Email addresses of authors:**

Maaz Anwer Memon: [maaz@student.usm.my](mailto:maaz@student.usm.my)

Noriko Mizusawa: [mizusawa@tokushima-u.ac.jp](mailto:mizusawa@tokushima-u.ac.jp)

Thirumulu Ponnuraj Kannan: [kannan@usm.my](mailto:kannan@usm.my)

Rizwan Mahmood: [rizwan96321@student.usm.my](mailto:rizwan96321@student.usm.my)

Khairul Mohd Fadzli Mustaffa: [khairulmf@usm.my](mailto:khairulmf@usm.my)

Muhammad Usman Ashraf: [usmankan@student.usm.my](mailto:usmankan@student.usm.my)

**Supplementary Data**

**Contents**

**1. Supplementary Tables**

*Supplementary Table S1. Details of QUADAS-2 quality assessment of each included study (QUADAS-2 tool).*

*Table S2: Variance–Covariance Matrices for miRNA Diagnostic Performance*

*Table S3: Influence / Leave-One-Out Table*

*Table S4: PPV / NPV at Plausible Prevalence Scenarios*

*Table S5: GRADE Table*

*Study-Level Diagnostic Accuracy Table (2×2 + Sens/Spec)*

**2. Appendix**

**3. Separate HSROC curves with 95% confidence regions for each miRNA**

**Supplementary Table S1.** Details of QUADAS-2 quality assessment of each included study (QUADAS-2 tool).

| **Appendix-1.** Search strategy (PubMed, Web of Science, Scopus, and Wiley Online Library).**Study** | **RISK OF BIAS** | | | | **APPLICABILITY CONCERNS** | | |
| --- | --- | --- | --- | --- | --- | --- | --- |
|  | **PATIENT SELECTION** | **INDEX TEST** | **REFERENCE STANDARD** | **FLOW AND TIMING** | **PATIENT SELECTION** | **INDEX TEST** | **REFERENCE STANDARD** |
| (Almiñana-Pastor et al., 2023) | \| 🟢 \| \| --- \| | \| 🟢 \| \| --- \| | \| 🟢 \| \| --- \| | \| 🟢 \| \| --- \| | \| 🟢 \| \| --- \| | \| 🟢 \| \| --- \| | \| 🟢 \| \| --- \| |
| (Yan et al., 2025) | \| 🟢 \| \| --- \| | \| 🟢 \| \| --- \| | \| 🟢 \| \| --- \| | \| 🟢 \| \| --- \| | \| 🟢 \| \| --- \| | \| 🟢 \| \| --- \| | \| 🟢 \| \| --- \| |
| (Li and Zhu, 2024) | \| 🟢 \| \| --- \| | \| 🟢 \| \| --- \| | \| 🟢 \| \| --- \| | \| 🟢 \| \| --- \| | \| 🟢 \| \| --- \| | \| 🟢 \| \| --- \| | \| 🟢 \| \| --- \| |
| (AbdelKawy et al., 2024) | \| 🟢 \| \| --- \| | \| 🟢 \| \| --- \| | \| 🟢 \| \| --- \| | \| 🟢 \| \| --- \| | \| 🟢 \| \| --- \| | \| 🟢 \| \| --- \| | \| 🟢 \| \| --- \| |
| (Baru et al., 2025) | \| 🟢 \| \| --- \| | \| 🟢 \| \| --- \| | \| 🟢 \| \| --- \| | \| 🟢 \| \| --- \| | \| 🟢 \| \| --- \| | \| 🟢 \| \| --- \| | \| 🟢 \| \| --- \| |
| (Appukuttan et al., 2025) | \| 🟢 \| \| --- \| | \| 🟢 \| \| --- \| | \| 🟢 \| \| --- \| | \| 🟢 \| \| --- \| | \| 🟢 \| \| --- \| | \| 🟢 \| \| --- \| | \| 🟢 \| \| --- \| |
| (Akshaya et al., 2025) | \| 🟢 \| \| --- \| | \| 🟢 \| \| --- \| | \| 🟢 \| \| --- \| | \| 🟢 \| \| --- \| | \| 🟢 \| \| --- \| | \| 🟢 \| \| --- \| | \| 🟢 \| \| --- \| |
| (Ibrahim et al., 2025) | \| 🟢 \| \| --- \| | \| 🟢 \| \| --- \| | \| 🟢 \| \| --- \| | \| 🟢 \| \| --- \| | \| 🟢 \| \| --- \| | \| 🟢 \| \| --- \| | \| 🟢 \| \| --- \| |
| (Bandi et al., 2024) | \| 🟢 \| \| --- \| | \| 🟢 \| \| --- \| | \| 🟢 \| \| --- \| | \| 🟢 \| \| --- \| | \| 🟢 \| \| --- \| | \| 🟢 \| \| --- \| | \| 🟢 \| \| --- \| |
| (Bandi et al., 2023a) | \| 🟢 \| \| --- \| | \| 🟢 \| \| --- \| | \| 🟢 \| \| --- \| | \| 🟢 \| \| --- \| | \| 🟢 \| \| --- \| | \| 🟢 \| \| --- \| | \| 🟢 \| \| --- \| |
| (Bandi et al., 2023b) | \| 🟢 \| \| --- \| | \| 🟢 \| \| --- \| | \| 🟢 \| \| --- \| | \| 🟢 \| \| --- \| | \| 🟢 \| \| --- \| | \| 🟢 \| \| --- \| | \| 🟢 \| \| --- \| |
| (Daily et al., 2023) | \| 🟢 \| \| --- \| | \| 🟢 \| \| --- \| | \| 🟢 \| \| --- \| | \| 🟢 \| \| --- \| | \| 🟢 \| \| --- \| | \| 🟢 \| \| --- \| | \| 🟢 \| \| --- \| |
| (Jiang et al., 2021) | \| 🟢 \| \| --- \| | \| 🟢 \| \| --- \| | \| 🟢 \| \| --- \| | \| 🟢 \| \| --- \| | \| 🟢 \| \| --- \| | \| 🟢 \| \| --- \| | \| 🟢 \| \| --- \| |
| (Motedayyen et al., 2015) | \| 🟢 \| \| --- \| | \| 🟢 \| \| --- \| | \| 🟢 \| \| --- \| | \| 🟢 \| \| --- \| | \| 🟢 \| \| --- \| | \| 🟢 \| \| --- \| | \| 🟢 \| \| --- \| |
| (Nandipati et al., 2022) | \| 🟢 \| \| --- \| | \| 🟢 \| \| --- \| | \| 🟢 \| \| --- \| | \| 🟢 \| \| --- \| | \| 🟢 \| \| --- \| | \| 🟢 \| \| --- \| | \| 🟢 \| \| --- \| |
| (Yoneda et al., 2019) | \| 🟢 \| \| --- \| | \| 🟢 \| \| --- \| | \| 🟢 \| \| --- \| | \| 🟢 \| \| --- \| | \| 🟢 \| \| --- \| | \| 🟢 \| \| --- \| | \| 🟢 \| \| --- \| |
| (Costantini et al., 2023) | \| 🟢 \| \| --- \| | \| 🟢 \| \| --- \| | \| 🟢 \| \| --- \| | \| 🟢 \| \| --- \| | \| 🟢 \| \| --- \| | \| 🟢 \| \| --- \| | \| 🟢 \| \| --- \| |
| (Yu, 2023) | \| 🟢 \| \| --- \| | \| 🟢 \| \| --- \| | \| 🟢 \| \| --- \| | \| 🟢 \| \| --- \| | \| 🟢 \| \| --- \| | \| 🟢 \| \| --- \| | \| 🟢 \| \| --- \| |
| (Zhu and Zhong, 2022) | \| 🟢 \| \| --- \| | \| 🟢 \| \| --- \| | \| 🟢 \| \| --- \| | \| 🟢 \| \| --- \| | \| 🟢 \| \| --- \| | \| 🟢 \| \| --- \| | \| 🟢 \| \| --- \| |
| (Baru et al., 2023) | \| 🟢 \| \| --- \| | \| 🟢 \| \| --- \| | \| 🟢 \| \| --- \| | \| 🟢 \| \| --- \| | \| 🟢 \| \| --- \| | \| 🟢 \| \| --- \| | \| 🟢 \| \| --- \| |
| (Huang and Jia, 2022) | \| 🟢 \| \| --- \| | \| 🟢 \| \| --- \| | \| 🟢 \| \| --- \| | \| 🟢 \| \| --- \| | \| 🟢 \| \| --- \| | \| 🟢 \| \| --- \| | \| 🟢 \| \| --- \| |
| (Ni et al., 2023) | \| 🟢 \| \| --- \| | \| 🟢 \| \| --- \| | \| 🟢 \| \| --- \| | \| 🟢 \| \| --- \| | \| 🟢 \| \| --- \| | \| 🟢 \| \| --- \| | \| 🟢 \| \| --- \| |
| (Du et al., 2021) | \| 🟢 \| \| --- \| | \| 🟢 \| \| --- \| | \| 🟢 \| \| --- \| | \| 🟢 \| \| --- \| | \| 🟢 \| \| --- \| | \| 🟢 \| \| --- \| | \| 🟢 \| \| --- \| |
| (Buragaite-Staponkiene et al., 2023) | \| 🟢 \| \| --- \| | 🟡 | \| 🟢 \| \| --- \| | \| 🟢 \| \| --- \| | \| 🟢 \| \| --- \| | \| 🟢 \| \| --- \| | \| 🟢 \| \| --- \| |
| (Elazazy et al., 2021) | \| 🟢 \| \| --- \| | 🟡 | \| 🟢 \| \| --- \| | \| 🟢 \| \| --- \| | \| 🟢 \| \| --- \| | \| 🟢 \| \| --- \| | \| 🟢 \| \| --- \| |
| (Liu et al., 2022) | \| 🟢 \| \| --- \| | 🟡 | \| 🟢 \| \| --- \| | \| 🟢 \| \| --- \| | \| 🟢 \| \| --- \| | \| 🟢 \| \| --- \| | \| 🟢 \| \| --- \| |
| (Radović et al., 2018) | 🟢 | 🟡 | \| 🟢 \| \| --- \| | \| 🟢 \| \| --- \| | 🟢 | \| 🟢 \| \| --- \| | \| 🟢 \| \| --- \| |
| (Yagnik et al., 2019) | 🔴 | 🟡 | \| 🟢 \| \| --- \| | \| 🟢 \| \| --- \| | \| 🔴 \| \| --- \| | \| 🟢 \| \| --- \| | \| 🟢 \| \| --- \| |
| (Rovas et al., 2021) | \| 🟢 \| \| --- \| | \| 🟡 \| \| --- \| | \| 🟢 \| \| --- \| | \| 🟢 \| \| --- \| | \| 🟢 \| \| --- \| | \| 🟢 \| \| --- \| | \| 🟢 \| \| --- \| |
| (Rovas et al., 2022) | \| 🟢 \| \| --- \| | \| 🟢 \| \| --- \| | \| 🟢 \| \| --- \| | \| 🟢 \| \| --- \| | \| 🟢 \| \| --- \| | \| 🟢 \| \| --- \| | \| 🟢 \| \| --- \| |
| (Wu et al., 2021) | \| 🟢 \| \| --- \| | 🟡 | \| 🟢 \| \| --- \| | \| 🟢 \| \| --- \| | \| 🟢 \| \| --- \| | \| 🟢 \| \| --- \| | \| 🟢 \| \| --- \| |
| (Wu et al., 2022) | \| 🟢 \| \| --- \| | \| 🟢 \| \| --- \| | \| 🟢 \| \| --- \| | \| 🟢 \| \| --- \| | \| 🟢 \| \| --- \| | \| 🟢 \| \| --- \| | \| 🟢 \| \| --- \| |

**Legend:**

🟢 **Green** = Low risk / Low concern

🟡 **Yellow** = Unclear risk / Unclear concern

🔴 **Red** = High risk / High concern

**Supplementary Table S2. Variance–Covariance Matrices for miRNA Diagnostic Performance**

| **miRNA** | **Parameter** | **logit Sensitivity** | **logit False-Positive Rate** |
| --- | --- | --- | --- |
| **miR-146** | logit Sensitivity | 1.4239 | −1.3757 |
|  | logit False-Positive Rate | −1.3757 | 1.4072 |
| **miR-155** | logit Sensitivity | 0.0765 | −0.0728 |
|  | logit False-Positive Rate | −0.0728 | 0.2413 |
| **miR-223** | logit Sensitivity | 0.1100 | −0.0295 |
|  | logit False-Positive Rate | −0.0295 | 0.0402 |

*Values rounded to 4 decimal places; derived from REML-estimated Reitsma models.*

*Between-study covariance estimates were extracted directly from the fitted Reitsma models using the variance–covariance (vcov) function in R. These matrices quantify the joint variability of sensitivity and specificity and were used for HSROC curve construction and heterogeneity interpretation*.

**Table S2: Influence / Leave-One-Out Table**

| **miRNA** | **Study Removed** | **logit(Sensitivity)** | **Sensitivity** | **logit(Specificity)** | **Specificity** | **Total Error (FN+FP)** | **Comment** | **Influential Flag** |
| --- | --- | --- | --- | --- | --- | --- | --- | --- |
| miR-146 | Alminana Pastor 2023 | 0.978 | 0.727 | 0.978 | 0.727 | 6 | Minimal change | No major influence |
| miR-146 | Radovic 2018 | ∞ | 1.000 | ∞ | 1.000 | 0 | Perfect accuracy | Influential |
| miR-146 | Rovas 2022 | 0.626 | 0.658 | 0.625 | 0.657 | 72 | Moderate change | No major influence |
| miR-155 | Appukuttan 2025 | 1.665 | 0.841 | -2.264 | 0.094 | 7 | Moderate effect | Potentially influential |
| miR-155 | Baru 2025 | 1.815 | 0.860 | -2.370 | 0.085 | 14 | Large FP effect | Potentially influential |
| miR-155 | Dailly 2023 | 1.577 | 0.829 | -1.762 | 0.146 | 6 | Slight effect | Potentially influential |
| miR-155 | Nandipati 2022 | 1.476 | 0.814 | -2.140 | 0.105 | 7 | Minimal effect | No major influence |
| miR-155 | Radovic 2018 | 1.738 | 0.850 | -1.758 | 0.146 | 12 | Small effect | Borderline |
| miR-223 | AbdelKawy 2024 | 0.842 | 0.698 | -1.131 | 0.244 | 9 | Moderate effect | Potentially influential |
| miR-223 | Bandi 2023a | 0.808 | 0.691 | -1.145 | 0.241 | 24 | Moderate effect | Potentially influential |
| miR-223 | Bandi 2023b | 0.929 | 0.716 | -1.127 | 0.244 | 11 | Slight effect | Potentially influential |
| miR-223 | Bandi 2024 | 0.734 | 0.676 | -1.169 | 0.236 | 21 | Moderate effect | Potentially influential |
| miR-223 | Elazazy 2021 | 0.658 | 0.659 | -1.177 | 0.236 | 10 | Minimal effect | No major influence |
| miR-223 | Liu 2022 | 1.097 | 0.750 | -1.526 | 0.178 | 46 | Large change, low Se & Sp | Influential |

*Leave-One-Out (LOO) sensitivity and specificity analysis for miR-146, miR-155, and miR-223. Sensitivity and specificity values are back-transformed from logit estimates. ∞ indicates a perfect sensitivity or specificity value (100%), which mathematically produces an infinite logit. Total Error = FN + FP per study removed. Influential studies were identified a priori based on: (i) sensitivity or specificity = 1.0, (ii) sensitivity or specificity < 0.50, or (iii) disproportionately high total error. These studies are flagged in the “Influential Flag” column. LOO analyses indicate the effect of removing each study on pooled diagnostic estimates.*

**Table S3: PPV / NPV at Plausible Prevalence Scenarios**

| **miRNA** | **Prevalence** | **PPV** | **PPV_Lower** | **PPV_Upper** | **NPV** | **NPV_Lower** | **NPV_Upper** |
| --- | --- | --- | --- | --- | --- | --- | --- |
| miR-146 | 0.05 | 0.252 | 0.050 | 0.050 | 0.992 | 0.950 | 0.950 |
| miR-146 | 0.10 | 0.416 | 0.100 | 0.100 | 0.983 | 0.900 | 0.900 |
| miR-146 | 0.20 | 0.616 | 0.200 | 0.200 | 0.963 | 0.800 | 0.800 |
| miR-146 | 0.50 | 0.865 | 0.499 | 0.500 | 0.866 | 0.500 | 0.500 |
| miR-155 | 0.05 | 0.270 | 0.431 | 0.153 | 0.990 | 0.993 | 0.986 |
| miR-155 | 0.10 | 0.438 | 0.615 | 0.276 | 0.980 | 0.985 | 0.971 |
| miR-155 | 0.20 | 0.637 | 0.782 | 0.461 | 0.956 | 0.967 | 0.938 |
| miR-155 | 0.50 | 0.875 | 0.935 | 0.774 | 0.843 | 0.879 | 0.790 |
| miR-223 | 0.05 | 0.139 | 0.189 | 0.103 | 0.980 | 0.985 | 0.974 |
| miR-223 | 0.10 | 0.255 | 0.329 | 0.195 | 0.959 | 0.969 | 0.946 |
| miR-223 | 0.20 | 0.435 | 0.525 | 0.353 | 0.911 | 0.932 | 0.886 |
| miR-223 | 0.50 | 0.755 | 0.815 | 0.686 | 0.720 | 0.774 | 0.660 |

**Table S4: GRADE Table**

| **miRNA** | **No. of Studies** | **Risk of Bias** | **Inconsistency** | **Indirectness** | **Imprecision** | **Publication Bias** | **Overall Certainty** |
| --- | --- | --- | --- | --- | --- | --- | --- |
| miR-146 | 3 | Not serious | Serious | Not serious | Serious | Not assessable | LOW ⬇⬇ |
| miR-155 | 5 | Not serious | Serious | Not serious | Not serious | Not serious | MODERATE ⬇ |
| miR-223 | 6 | Not serious | Serious | Not serious | Not serious | Serious | LOW ⬇⬇ |

**Study-Level Diagnostic Accuracy Table (2×2 + Sens/Spec)**

| **miRNA** | **Study** | **TP** | **FN** | **FP** | **TN** | **Sensitivity** | **Specificity** |
| --- | --- | --- | --- | --- | --- | --- | --- |
| miR-146 | Alminana Pastor 2023 | 8 | 3 | 3 | 8 | 0.727 | 0.727 |
| miR-146 | Radovic 2018 | 48 | 0 | 0 | 48 | 1.000 | 1.000 |
| miR-146 | Rovas 2022 | 50 | 26 | 46 | 88 | 0.658 | 0.657 |
| miR-155 | Appukuttan 2025 | 20 | 4 | 3 | 9 | 0.833 | 0.750 |
| miR-155 | Baru 2025 | 12 | 5 | 9 | 24 | 0.706 | 0.727 |
| miR-155 | Dailly 2023 | 54 | 6 | 0 | 60 | 0.900 | 1.000 |
| miR-155 | Nandipati 2022 | 46 | 3 | 4 | 45 | 0.939 | 0.918 |
| miR-155 | Radovic 2018 | 46 | 10 | 2 | 38 | 0.821 | 0.950 |
| miR-223 | AbdelKawy 2024 | 20.5 | 8.5 | 0.5 | 14.5 | 0.707 | 0.967 |
| miR-223 | Bandi 2023a | 37 | 13 | 11 | 39 | 0.740 | 0.780 |
| miR-223 | Bandi 2023b | 15 | 10 | 1 | 24 | 0.600 | 0.960 |
| miR-223 | Bandi 2024 | 40 | 10 | 11 | 39 | 0.800 | 0.780 |
| miR-223 | Elazazy 2021 | 35 | 5 | 5 | 15 | 0.875 | 0.750 |
| miR-223 | Liu 2022 | 19 | 29 | 17 | 32 | 0.396 | 0.653 |

**2. Appendix**

**The rationale of the search strategy:**

The chosen keywords aim to capture relevant literature on the association between periodontal disease and microRNA (miRNA), specifically focusing on aspects related to diagnosis and biomarkers. Detailed search strategies were tailored to each database, utilizing specific keywords and Boolean connectors (AND, OR). The search was restricted to articles published up to 31^st^ May 2025.

**Search strategy**

**Database: PubMed (Search date: 31/05/2025)**

**#Periodontal Disease Terms**

1. "periodontal disease"[MeSH Terms] = 101,128
2. "periodontal disease"[All fields] = 117,284
3. "periodontics"[ All fields] = 139,422
4. "periodontitis"[ All fields] = 139,422
5. (1 OR 2 OR 3 OR 5) = 166,186

**#Biological Marker Terms**

1. "biomarker"[MeSH Terms] = 950,138
2. "biomarker"[All fields] = 1,272,581
3. "bio-marker"[All fields] = 468
4. "diagnosis"[MeSH Terms] = 9,824,276
5. “diagnosis"[ All fields] = 12,248,263
6. (6 OR 7 OR 8 OR 9 OR 10) = 12,794,343

**#miRNA Terms**

1. "microRNA"[MeSH Terms] = 142,415
2. microRNA"[All fields Terms]= 181,428
3. "miRNA"[ All fields] =184,896
4. "miRNAs"[All fields]= 184,896
5. (12 OR 13 OR 14 OR 15) =197,658

**(5 AND 11 AND 16) = 246**

("periodontal diseases"[MeSH Terms] OR ("periodontal diseases"[MeSH Terms] OR ("periodontal"[All Fields] AND "diseases"[All Fields]) OR "periodontal diseases"[All Fields] OR ("periodontal"[All Fields] AND "disease"[All Fields]) OR "periodontal disease"[All Fields]) OR ("periodontal"[All Fields] OR "periodontally"[All Fields] OR "periodontically"[All Fields] OR "periodontics"[MeSH Terms] OR "periodontics"[All Fields] OR "periodontic"[All Fields] OR "periodontitis"[MeSH Terms] OR "periodontitis"[All Fields] OR "periodontitides"[All Fields]) OR ("periodontal"[All Fields] OR "periodontally"[All Fields] OR "periodontically"[All Fields] OR "periodontics"[MeSH Terms] OR "periodontics"[All Fields] OR "periodontic"[All Fields] OR "periodontitis"[MeSH Terms] OR "periodontitis"[All Fields] OR "periodontitides"[All Fields])) AND ("biomarkers"[MeSH Terms] OR ("biomarker s"[All Fields] OR "biomarkers"[MeSH Terms] OR "biomarkers"[All Fields] OR "biomarker"[All Fields]) OR (("mater today bio"[Journal] OR "biosci bioeng"[Journal] OR "biol open"[Journal] OR "bio"[All Fields]) **AND** ("marker"[All Fields] OR "markers"[All Fields])) OR "diagnosis"[MeSH Terms] OR ("diagnosable"[All Fields] OR "diagnosi"[All Fields] OR "diagnosis"[MeSH Terms] OR "diagnosis"[All Fields] OR "diagnose"[All Fields] OR "diagnosed"[All Fields] OR "diagnoses"[All Fields] OR "diagnosing"[All Fields] OR "diagnosis"[MeSH Subheading])) **AND** ("micrornas"[MeSH Terms] OR ("microrna s"[All Fields] OR "micrornas"[MeSH Terms] OR "micrornas"[All Fields] OR "microrna"[All Fields]) OR ("micrornas"[MeSH Terms] OR "micrornas"[All Fields] OR "mirna"[All Fields] OR "mirnas"[All Fields] OR "mirna s"[All Fields]) OR ("micrornas"[MeSH Terms] OR "micrornas"[All Fields] OR "mirna"[All Fields] OR "mirnas"[All Fields] OR "mirna s"[All Fields]))=**246**

**Database: Wiley Online Library (Search date: 31/05/2025)**

"periodontal disease OR periodontics OR periodontitis AND biomarker OR bio-marker OR diagnosis AND microRNA OR miRNA OR miRNAs" in Title= **88**

**Database: ISI Web of Science (Search date: 31/05/2025)**

((ALL=(periodontal disease OR periodontics OR periodontitis)) AND ALL=(biomarker OR bio-marker OR diagnosis)) AND ALL=(microRNA OR miRNA OR miRNAs)= 90

**Database: Scopus (Search date: 31/05/2025)**

( TITLE-ABS-KEY ( periodontal AND disease OR periodontics OR periodontitis ) AND TITLE-ABS-KEY ( biomarker OR bio-marker OR diagnosis ) AND TITLE-ABS-KEY ( microRNA OR miRNA OR miRNAs ) )= **128**


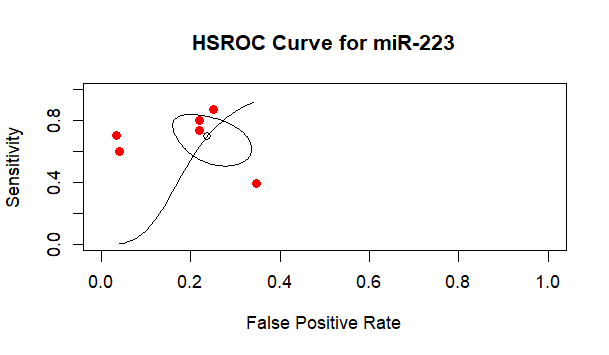

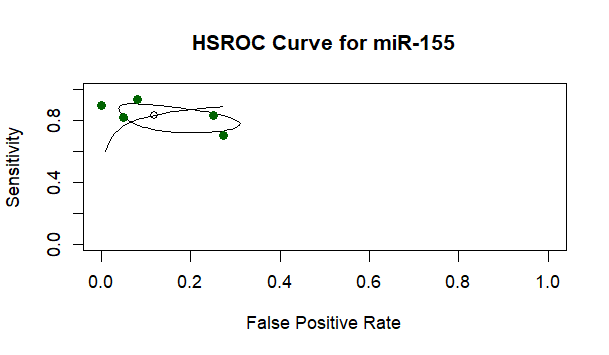

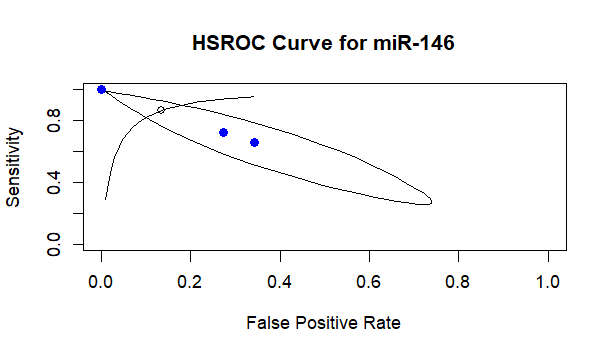

Supplement: Supplementary file 1 — Supplementary file1 (DOCX 87 KB) [file 10266_2025_1299_MOESM1_ESM.docx]
